# P-1802. Mortality in Hospitalised Patients with Dengue Fever: An Ambispective Observational Study

**DOI:** 10.1093/ofid/ofaf695.1971

**Published:** 2026-01-11

**Authors:** Bollapally Naveen Goud, Atul Gogia, Richa Dewan, Atul Kakar

**Affiliations:** Sir Ganga Ram Hospital, New Delhi, Delhi, India; Sir Ganga Ram Hospital, New Delhi, India, NEW DELHI, Delhi, India; Sir Ganga Ram Hospital, New Delhi, Delhi, India; Sir Ganga Ram Hospital, New Delhi, Delhi, India

## Abstract

**Background:**

Dengue fever, a major vector-borne illness in India, presents a wide clinical spectrum ranging from mild febrile illness to severe complications like shock and organ dysfunction. Hemophagocytic lymphohistiocytosis (HLH), a hyper-inflammatory state, has emerged as a potential contributor to mortality in severe dengue, though its recognition and clinical impact remain underexplored in adult Indian population.

**Methods:**

This ambispective observational study was conducted at Sir Ganga Ram Hospital between January 2022 and August 2024. A total of 276 adult dengue patients (123 prospective, 153 retrospective) were categorized based on WHO 2009 criteria into dengue without warning signs, dengue with warning signs, and severe dengue. HLH was diagnosed using adapted HLH-2004 criteria. Demographic, clinical, and laboratory data were collected.

**Results:**

Of the 276 patients, 135 (48.9%) had severe dengue. All 26 recorded deaths and 4 LAMA cases occurred within the severe dengue subgroup. Among severe dengue cases, 33 (24.4%) were diagnosed with HLH. The gender-wise severity analysis showed highest severe dengue prevalence in female prospective (61%) and male retrospective (51.2%) groups. Mean AST and ferritin levels were significantly higher in non-survivors (AST: 17,940 vs 5,851 U/L, p=0.036; Ferritin: 44,846 vs 8,556 ng/mL, p=0.0018). ALT did not significantly differ. While HLH subgroup had higher inflammation markers, its mortality (18%) was not statistically different from non-HLH severe dengue (17%) (p=0.603).

Complications strongly associated with mortality included:

• Fluid refractory hypotension

• MODS (p< 0.0001)

• Severe bleeding requiring transfusion

• HLH

**Conclusion:**

Severe dengue remains the only category associated with in-hospital mortality. Gender-specific severity patterns were noted, particularly among female prospective and male retrospective groups. Elevated AST and ferritin levels are significant mortality predictors. Although HLH did not independently increase mortality risk, its associated complications emphasize the importance of early detection and immunomodulatory therapy. These findings advocate for the incorporation of HLH screening protocols in severe dengue management to improve clinical outcomes

**Disclosures:**

All Authors: No reported disclosures

